# Vertebral fracture risk in patients with differentiated thyroid cancer receiving TSH-suppressive therapy

**DOI:** 10.1530/EC-26-0103

**Published:** 2026-07-17

**Authors:** Dilek Gogas Yavuz, Ceyda Dincer Yazan, Zeliha Hekimsoy, Kadriye Aydin, Naile Gokkaya, Canan Ersoy, Berna İmge Aydoğan, Aysen Akalin, Ömercan Topaloğlu, Esra Nur Ademoglu Dilekci, Guven Baris Cansu, Bulent Can, Kagan Gungor, Mehtap Evran, Lezzan keskin, Ziynet Alphan, Özlem Turhan İyidir, Levent Ozsari, Tayfun Garip, Gulsah Elbuken, Meral Mert, Zafer Pekkolay, Zeynep Cantürk, Can Ilgin, Onur Buğdaycı, Nilufer Ozdemir, Goknur Yorulmaz, Ahmet Toygar Kalkan, Yasemin Aydogan Unsal, Adnan Yay, Baris Karagun, Evin Bozkur

**Affiliations:** ^1^ Marmara University School of Medicine, Department of Endocrinology and Metabolism, Istanbul, Turkey; ^2^ Celal Bayar University, School of Medicine, Department of Endocrinology and Metabolism, Manisa, Turkey; ^3^ Lutfi Kirdar Research and Training Hospital, Endocrinology and Metabolism, Istanbul, Turkey; ^4^ Uludag University School of Medicine, Department of Endocrinology and Metabolism, Bursa, Turkey; ^5^ Batman Research and Training Hospital, Endocrinology and Metabolism, Batman, Turkey; ^6^ Osmangazi University School of Medicine, Department of Endocrinology and Metabolism, Eskisehir, Turkey; ^7^ Kocaeli Derince Research and Training Hospital, Endocrinology and Metabolism, Kocaeli, Turkey; ^8^ Bolu Abant Izzet Baysal University School of Medicine, Department of Endocrinology and Metabolism, Bolu, Turkey; ^9^ Kutahya Health Sciences University, Endocrinology and Metabolism, Kutahya, Turkey; ^10^ Medeniyet University School of Medicine, Department of Endocrinology and Metabolism, Istanbul, Turkey; ^11^ Cukurova University School of Medicine, Department of Endocrinology and Metabolism, Adana, Turkey; ^12^ Malatya Turgut Ozal University Faculty of Medicine, Endocrinology and Metabolism, Malatya, Turkey; ^13^ Usak Research and Training Hospital, Endocrinology and Metabolism, Usak, Turkey; ^14^ Baskent University School of Medicine, Department of Endocrinology and Metabolism, Ankara, Turkey; ^15^ Sultan Abdulhamid Han Research and Training Hospital, Endocrinology and Metabolism, Istanbul, Turkey; ^16^ Medical Park Pendik Hospital, Endocrinology and Metabolism, Istanbul, Turkey; ^17^ Namik Kemal University, School of Medicine, Department of Endocrinology and Metabolism, Tekirdag, Turkey; ^18^ Bakirkoy Sadi Konuk Research and Training Hospital, Endocrinology and Metabolism, Istanbul, Turkey; ^19^ Dicle University School of Medicine, Department of Endocrinology and Metabolism, Diyarbakir, Turkey; ^20^ Kocaeli University School of Medicine, Endocrinology and Metabolism, Kocaeli, Turkey; ^21^ Marmara University Faculty of Medicine, Radiation Oncology, Istanbul, Turkey; ^22^ Marmara University School of Medicine, Department of Radiology, Istanbul, Turkey

**Keywords:** bone, fracture, differentiated thyroid cancer, TSH suppression

## Abstract

**Background:**

Suppression of thyroid-stimulating hormone (TSH) can adversely affect bone tissue in individuals with differentiated thyroid cancer (DTC). This study aims to examine bone mineral density (BMD) and vertebral fractures (VFs) in DTC patients receiving levothyroxine (LT4) suppression therapy.

**Methods:**

A total of 990 patients with DTC who had undergone total thyroidectomy and received levothyroxine suppression therapy for a minimum of one year were included in the study. Clinical and laboratory data obtained at the most recent visit were extracted from patient records. BMD measurements of the lumbar spine and femoral regions, assessed by dual-energy X-ray absorptiometry (DXA) within the preceding year, were recorded. When available, lateral vertebral radiographs were also evaluated.

**Results:**

The study included 990 patients with DTC. According to DXA results, 545 (55.2%) had normal BMD, 363 (36.6%) had osteopenia, and 82 (8.2%) had osteoporosis. Osteoporotic patients were older and had lower BMI (*P* = 0.001 for both), while TSH levels were similar across groups. Among 476 patients with vertebral radiographs, 131 (27.5%) had moderate-to-severe VFs. Fracture rates were significantly higher in patients with severe TSH suppression compared with other groups (*P* = 0.013). Patients with VFs were older and had lower femoral neck and L1–L4 BMD (*P* = 0.001). Regression analysis showed that disease duration, FT4 levels, and femoral neck BMD were associated with fracture risk (*P* = 0.001). Multivariate analysis identified age, femoral neck BMD, L1–L4 BMD, and osteocalcin as independent predictors of osteoporosis (*P* = 0.001). TSH subgroups showed no differences in BMD values.

**Conclusion:**

Findings suggest that DTC patients undergoing TSH suppression have a significant risk of VFs. Assessments of VFs should be integrated into routine care for DTC survivors to ensure long-term skeletal safety.

## Introduction

Thyroid-stimulating hormone (TSH) suppression treatment is usually recommended for differentiated thyroid cancer (DTC) patients following thyroidectomy to reduce the risk of recurrence and mortality for those at intermediate and high risk, as determined by their postoperative response status according to American Thyroid Association (ATA) 2015 guidelines (1, 2).

Recent evidence indicates that TSH over-suppression is a common clinical concern potentially associated with bone complications (3). Endogen hyperthyroidism was shown in large-scale cohort studies to have detrimental effects on bone remodeling, accelerate bone turnover, and reduce bone mineral density (BMD) (4). ATA 2016 hyperthyroidism management guidelines point to the increased osteoporosis and fracture risk and strongly recommend treatment of patients with TSH < 0.1 mIU/L (5).

A recent meta-analysis indicates that subclinical hyperthyroidism is associated with an elevated risk of hip and spine fractures (6). The studies on BMD and fracture risk involved a limited cohort of DTC patients, yielding inconsistent results due to gender differences and variability within the study populations. The effects of iatrogenic hyperthyroidism on BMD and fracture risk are controversial in DTC patients. According to a case–control study that included both a control group and patients with thyroid cancer, U.S. veterans with thyroid cancer had a higher risk of osteoporosis than controls, but not of fractures. Researchers found that lower TSH levels, female sex, advanced age, and androgen use are associated with osteoporosis. TSH levels showed no correlation with fracture incidence (7). Two meta-analyses demonstrated deleterious effects on BMD levels in postmenopausal patients undergoing TSH suppression therapy (8, 9).

In a Korean study, osteoporosis was found to be higher in the group of patients with thyroid cancer, but fracture status was comparable with controls (10). A small cohort of DTC patients was shown to have no increased fracture risk compared with the healthy population (11). In another study, long-term suppression with levothyroxine in men with DTC does not appear to have a negative effect on BMD or increase the incidence of vertebral fractures (VFs) (12).

A European study indicated a high prevalence of VFs among women with DTC undergoing long-term, suppressive LT4 treatment (13). A recent study indicated that the prevalence of VFs was significantly higher in the DTC cohort (44.1%) compared with healthy controls in the Japan DTC group (14).

The primary aim of this multicenter, nationwide study was to determine the prevalence of osteoporosis, osteopenia, and VFs and to identify the clinical determinants of VFs in patients with DTC receiving levothyroxine (LT4) suppression therapy following total thyroidectomy, with or without radioiodine ablation.

## Materials and methods

This cross-sectional study was conducted at 21 medical centers in 12 cities (Istanbul, Kocaeli, Manisa, Bursa, Eskisehir, Malatya, Diyarbakir, Adana, Usak, Batman, Bolu, and Tekirdag) in different regions of Turkey. The study was approved by the Local Ethics Committee of Marmara University (09.2017.359) and conducted in compliance with the Declaration of Helsinki. Laboratory values from the most recent visit and BMD measurements and vertebral X-rays taken within the previous year were obtained from files, and data were collected between 2018 and 2020.

### Patient selection

A total of 1,100 DTC patients were screened for this study. Of these, 990 patients aged over 18 years who had been diagnosed with DTC, had undergone total thyroidectomy (with or without a history of radioactive iodine (RAI) therapy), had received levothyroxine suppression therapy for at least one year, and had available BMD measurements for both the lumbar spine and femoral regions were included. In addition, a subgroup of 476 patients with available vertebral radiographs was included for further analysis (Supplement 1 (see section on Supplementary materials given at the end of the article)).

Participants with conditions known to affect bone metabolism – such as central hypothyroidism, primary hyperparathyroidism, inflammatory bowel disease, malabsorption syndromes, a history of gastric bypass surgery, other malignancies, bone metastases, and medullary or anaplastic thyroid carcinoma – were excluded from the study. In addition, individuals receiving medications that could influence bone metabolism, including glucocorticoids, antiepileptic drugs, or hormone replacement therapy, were not eligible for inclusion. Participants currently undergoing triiodothyronine (T3) therapy were also excluded from the study.

### Clinical evaluation

Clinical data, including age, sex, disease duration, radioactive iodine treatment, and comorbid diseases such as hypertension, diabetes mellitus, cerebrovascular disease, coronary artery disease, atrial fibrillation, malabsorption, inflammatory bowel disease, and gluten enteropathy, were extracted from medical records.

Data from pathology reports were extracted from files. The daily doses of LT4 were documented. The quantity of LT4 per kilogram was determined.

Body weight and height were measured, and BMI (body mass index) was calculated as kg/m^2^ using the BMI formula. After 15 min of rest, the systolic and diastolic blood pressures and heart rate were measured.

### Laboratory evaluation

Serum TSH, FT4, serum thyroglobulin (Tg), and anti-thyroglobulin (anti-Tg) levels have been recorded from patients’ files within three months. The mean level of the current, 6-month-ago, and 1-year-ago TSH levels was calculated for each patient. The third-generation chemiluminescence immunoassay method was employed to quantify thyroid hormones in hospital laboratories, where patient monitoring was also conducted. The laboratory reference ranges for TSH and FT4 were similar. The reference range for TSH is 0.34–5.60 mU/L, while FT4 ranges from 0.61 to 1.12 ng/dL (0.144–0.26 pmol/L). The minimum detectable level of TSH was 0.01 mIU/L. Venous blood samples for thyroid function analysis were collected after an 8 h fast, prior to the ingestion of the morning pill.

Levels of serum calcium, phosphorus, parathormone, 25-hydroxyvitamin D3, osteocalcin, and C-telopeptide were documented in patient files during the latest visit 25(OH)D levels were assessed utilizing the Beckman Coulter paramagnetic particle chemiluminescence immunoassay technique. The serum intact parathyroid hormone levels were measured using an electrochemiluminescence immunoassay on the Cobas 6000 analyzer (Roche Diagnostics, Germany) with a reference range of 15–65 ng/L. Inorganic phosphorus (reference range: 2.6–4.5 mg/dL) and calcium (reference range: 8.8–10.6 mg/dL) were measured photometrically (Beckman Coulter, USA), whereas osteocalcin (3–13 μg/L) and C-telopeptide (0.02–0.57 ng/mL) were measured by chemiluminescence immunoassay.

The ATA 2015 management guidelines for adult thyroid cancer patients recommend maintaining target TSH levels at 0.1 mU/L for high-risk individuals, between 0.1 and 0.5 mU/L for those at intermediate risk, and between 0.5 and 2 for low-risk patients i). Patients’ TSH levels were classified as severely suppressed (TSH: 0.01 mU/L), moderately suppressed (TSH: 0.01–0.1 mU/L), mildly suppressed (TSH: 0.1–0.5 mU/L), or non-suppressed (TSH > 0.5 mU/L) according to ranges defined in the ATA 2015 guidelines. Patients were also classified based on their achievement of target TSH levels, which were adjusted for characteristics such as disease duration, risk, and response status. This classification differentiated between patients who achieved the target levels and those who did not.

### Bone mineral density evaluation

BMD was assessed by dual-energy X-ray absorptiometry (DXA) at all centers within one year, in accordance with International Society for Clinical Densitometry (ISCD) recommendations. Measurements were obtained from the lumbar spine (L1–L4) in the anteroposterior (AP) projection and from three regions of the right hip, including the femoral neck, using predominantly GE Lunar systems. All devices underwent regular calibration and quality control procedures, and BMD reports were reviewed to ensure adherence to standardized acquisition techniques. Repeated measurements demonstrated acceptable precision, with 68% of values falling within one standard deviation (SD) (0.012 g/cm^3^), consistent with expected DXA variability. Osteopenia and osteoporosis were classified in accordance with the standards established by the World Health Organization, which rely on T and Z scores. Osteopenia is defined as a T score lying within the range of −1 SD to −2.5 SD for either the lumbar anterior–posterior (AP) or femoral neck. Osteoporosis is characterized by a BMD T score, evaluated using dual-energy X-ray absorptiometry (DXA), that falls at or below −2.5 SDs from the mean at either the lumbar spine or femoral neck. This diagnostic criterion applies exclusively to postmenopausal women and males who are 50 years of age or older. This criterion defines low BMD for premenopausal women and males under the age of 50 as a BMD Z score on DXA at the lumbar spine or femoral neck that is less than or equal to −2 SD, categorizing them within the osteoporotic group based on chronological age (15).

### Vertebral fracture evaluation

A total of 476 patients had vertebral X-rays. VFs were assessed by lateral thoracolumbar X-ray radiograms, which were performed independently by two specialists. The semi-quantitative technique developed by Genant *et al.* was applied (16). The grading system divided fractures into four categories: normal (grade 0); mild (grade 1), which involved a reduction of approximately 20–25% in anterior, middle, and/or posterior height and a reduction of area by 10–20%; and moderate (grade 2), which involved a reduction of approximately 25–40% in any height and a reduction of area by 20–40%; and severe (grade 3), which involved a reduction of approximately 40% in any height and area. Vertebral radiograms were independently evaluated by two blinded specialists (O B and D G Y).

### Statistical analysis

The distribution of the data was evaluated by the Shapiro–Wilk test. A one-way ANOVA test was employed for comparing the data demonstrating normal distribution among the three groups. The Kruskal–Wallis ANOVA test was used to compare variables that did not have a normal distribution among the three groups. The Fisher exact test examined the differences between categorical data sets. Post hoc comparisons of the variables found meaningful after the ANOVA and Kruskal–Wallis tests analyzed Dunn’s test. Numerical variables are expressed as mean ± SD, and categorical variables as frequency (percentage). Bonferroni correction was applied in multiple group analyses. The significance level for subgroup analysis was *P* < 0.0167 for three parameters and *P* < 0.0083 for four parameters. Variables included in the multivariate analysis were selected based on clinical relevance and significance in univariate analysis: age, sex, disease duration, mean daily LT4 dose/kg, menopausal status, femur neck BMD, L1–L4 BMD, C-telopeptide, and osteocalcin were the variables for osteoporosis and age, sex, duration of disease, mean daily levothyroxine dose/kg, menopausal status, femur neck BMD, L1–L4 BMD, and FT4 levels were the variables for fracture risk. Potential confounders and multicollinearity were also assessed before inclusion in the final model. All analyses were executed using Stata 15.1 software (StataCorp, USA).

## Results

### Clinical characteristics of the patients

There were 990 DTC patients enrolled (F/M: 829/161), with a mean age of 51.4 ± 11.5 years and a mean disease duration of 5.2 ± 4.4 years. The average daily intake of LT4 was 131 ± 37.5 μg/day. Table 1 presents the clinical and laboratory features of the study group categorized by gender. The mean TSH level within one year of the overall study group was 1.94 ± 8.02 mU/L. The mean femur neck BMD was 0.930 ± 0.18 g/cm^2^, while the L1–L4 BMD was 1.070 ± 0.204 g/cm^2^. Women were younger (*P* = 0.037) and had a higher BMI (*P* < 0.001) than men. Women had a lower daily total LT4 dosage (*P* < 0.001), LT4 dose/kg (*P* < 0.001), BMD at L1–L4 (*P* < 0.001), and femur neck BMD (*P* < 0.001) than men. TSH and FT4 levels were similar according to gender. Men had greater serum calcium (*P* < 0.001) and 25(OH)D levels (*P* = 0.001) than women. Thyroid cancer types and radioiodine therapy details are provided in Supplement 2.

**Table 1:** Clinical and laboratory characteristics of patients with differentiated thyroid cancer.

	Total (*n* = 990)	Men (*n* = 161)	Women (*n* = 829)	*P* *
Age (years)	51.4 ± 11.5	53.3 ± 12.5	51.07 ± 11.2	0.037
Duration of disease (years)	5.2 ± 4.4	4.85 ± 4.64	5.2 ± 4.4	0.13
BMI (kg/m^2^)	30.5 ± 5.69	28.9 ± 4.22	30.8 ± 5.89	<0.001
SBP (mmHg)	125.6 ± 19	129.2 ± 19.2	125 ± 18.9	0.018
DBP (mm/Hg)	77.7 ± 11.89	76.2 ± 19.2	77.4 ± 11.7	0.12
Pulse (per minute)	79.6 ± 10.2	82 ± 12.3	79.2 ± 9.83	0.051
LT4 dose μg/kg	1.69 ± 0.47	1.67 ± 0.48	1.79 ± 0.45	<0.001
LT4 daily dosage (μg/day)	131 ± 37.5	152.2 ± 41.3	126.9 ± 35.3	<0.001
Femur neck BMD (g/cm^2^)	0.930 ± 0.18	1.000 ± 0.18	0.925 ± 0.18	<0.001
Femur neck T score	−0.31 ± 1.24	−0.23 ± 1.32	−0.3 ± 1.23	0.41
Femur neck Z score	0.29 ± 1.14	0.38 ± 1.35	0.28 ± 1.105	0.17
L1–L4 BMD (g/cm^2^)	1.070 ± 0.204	1.130 ± 0.22	1.060 ± 0.19	<0.001
L1–L4 T score	−0.44 ± 1.63	−0.07 ± 1.73	−0.51 ± 1.60	0.0038
L1–L4 Z score	−0.005 ± 1.43	0.18 ± 1.75	−0.04 ± 1.36	0.23
TSH (mU/L)	1.94 ± 8.02	1.73 ± 4.43	1.99 ± 8.54	0.71
FT4 (ng/dL)	3.38 ± 4.91	3.3 ± 4.87	3.4 ± 4.92	0.77
Calcium (mg/dL)	9.47 ± 0.59	9.6 ± 0.52	9.45 ± 0.60	<0.001
Phosphorus (mg/dL)	3.59 ± 0.72	3.3 ± 0.65	3.65 ± 0.72	<0.001
Parathormone (ng/dL)	52.5 ± 37.5	53.1 ± 36.8	51.8 ± 33.5	0.66
25(OH)D (ng/dL)	22.45 ± 12.9	24.8 ± 11.7	22.0 ± 13.09	0.001
Osteocalcin (ng/mL)	5.11 ± 4.29	4.8 ± 3.5	5.1 ± 4.4	0.81
C-telopeptide (ng/mL)	0.86 ± 5.85	0.75 ± 0.6	0.88 ± 0.75	0.90

* *p* values represent comparisons between female and male groups.

### Assessment of bone mineral density

BMD evaluation revealed that 545 patients (55.2%) have normal BMD, 82 individuals (8.2%) have osteoporosis, and 363 patients (36.6%) had osteopenia.

The patients with normal BMD were younger than the osteopenic (*P* < 0.001) and osteoporotic patients (*P* = 0.005). The BMI of the osteoporotic/low bone mass group was significantly lower than that of the osteopenic (*P* < 0.001) and the normal group (*P* < 0.001). The osteoporotic group had a higher serum PTH than the osteopenic and normal BMD groups (*P* < 0.001). The serum 25(OH)D levels in the groups were similar. Patients with osteoporosis had higher osteocalcin levels than the normal group (*P* < 0.001). Laboratory and clinical characteristics of the osteoporotic, osteopenic, and normal BMD groups are shown in Table 2.

**Table 2:** Clinical and laboratory values of patients according to osteoporosis status.

	Osteoporosis	Osteopenia	Normal BMD	*P*
	(*n* = 82)	(*n* = 363)	(*n* = 545)	
Age (years)	57.5 ± 9.14	55.8 ± 10.1	47.5 ± 11.2	<0.001
Duration of disease (years)	5.5 ± 4.93	5.5 ± 4.9	4.94 ± 3.9	0.50
BMI (kg/m^2^)	28.4 ± 4.82	30.3 ± 5.13	30.9 ± 6.1	<0.001
SBP (mmHg)	126.4 ± 18.2	127.9 ± 20.89	123.8 ± 17.4	0.03
DBP (mm/Hg)	78.7 ± 11.6	78 ± 12.3	77.3 ± 11.6	0.63
Pulse (per minute)	78.06 ± 9.07	79.3 ± 10.23	80.1 ± 10.45	0.16
LT4 dose (μg/kg)	122.2 ± 35.06	122.3 ± 36.3	138.3 ± 37.1	<0.001
LT4 daily dosage (μg/day)	122.2 ± 35.06	122.3 ± 36.3	138.3 ± 37.1	<0.001
Femur neck BMD (g/cm^2^)	0.78 ± 0.14	0.84 ± 0.12	1.02 ± 0.18	<0.001
Femur neck T-score	−1.5 ± 1.08	−1.02 ± 0.93	0.36 ± 1.02	<0.001
Femur neck Z-score	−0.6 ± 1.26	−0.19 ± 0.92	0.76 ± 1.04	<0.001
L1–L4 BMD (g/cm^2^)	0.79 ± 0.1	0.977 ± 0.125	1.18 ± 0.18	<0.001
L1–L4 T score	−2.8 ± 0.63	−1.3 ± 0.77	0.52 ± 1.44	<0.001
L1–L4 Z score	−1.87 ± 0.83	−0.66 ± 0.911	0.71 ± 1.33	<0.001
Mean TSH within one year (mU/L)	1.58 ± 5.16	1.86 ± 6.7	2.05 ± 9.1	0.501
FT4 (ng/dL)	3.6 ± 5.2	4 ± 5.44	2.95 ± 4.44	0.001
Calcium (mg/dL)	9.5 ± 0.47	9.56 ± 0.55	9.40 ± 0.63	0.0012
Phosphorus (mg/dL)	3.6 ± 0.57	3.6 ± 0.79	3.58 ± 0.69	0.33
Parathormone (ng/dL)	59.9 ± 30	55 ± 33.4	48.8 ± 34.7	<0.001
25(OH)D (ng/dL)	21.4 ± 13.8	22.5 ± 13.5	22.5 ± 12.3	0.51
Osteocalcin (ng/mL)	7.9 ± 7.9	5.2 ± 3.86	4.61 ± 3.67	<0.001

### Assessment of vertebral fracture

A total of 476 patients were evaluated using the lateral spinal X-ray. A total of 200 patients (42.0%) displayed a minimum of a grade 1 fracture, and 131 (27.5%) had moderate/severe VFs during the assessment of spinal X-rays.

Seventeen of the 131 patients with moderate–severe fractures were premenopausal or male under 50 years. A total of 193 patients exhibited the presence of at least one thoracic VF, whereas 87 patients displayed the occurrence of at least one lumbar VF. Table 3 displays the clinical and laboratory data of patients according to VF status. The age of the individuals with fractures was significantly higher (*P* < 0.001) compared with the non-fractured group. There were no significant differences seen in the daily dosage (*P* = 0.1) and dose per kg (*P* = 0.13) between the groups. The femur neck BMD of the fracture group was significantly lower compared with patients without fractures (*P* = 0.0001). In addition, the L1–L4 BMD of the fracture group was also significantly lower than that of patients without fractures (*P* = 0.0001). Patients with fractures had higher TSH levels (*P* = 0.0088) than those without fractures.

**Table 3:** DTC patients’ clinical and laboratory characteristics by status of vertebral fracture.

	Patients with fracture (*n* = 131)	Patients without fracture (*n*-345)	*P*
Age (years)	55.05 ± 10.7	50.22 ± 11.04	<0.001
Duration of disease (years)	6.20 ± 5.29	5.07 ± 3.73	0.054
BMI (kg/m^2^)	31.1 ± 5.17	30.7 ± 5.68	0.25
LT4 daily dose (μg/kg)	1.64 + 0.46	1.70 ± 0.47	0.1
LT4 daily dosage (μg/day)	127.6 ± 34.9	133.2 ± 38.6	0.13
Femur neck BMD (gr/cm^2^)	0.907 ± 0.13	0.970 ± 0.16	0.0001
Femur neck T score	−0.68 ± 1.1	−0.259 ± 1.235	0.0012
Femur neck Z score	−0.68 ± 1.13	0.218 ± 1.043	0.034
L1–L4 BMD (gr/cm^2^)	1.043 ± 0.16	1.135 ± 0.203	0.0001
L1–L4 T score	−0.934 ± 1.17	−0.108 ± 1.861	0.0001
L1–L4 Z score	−0.438 ± 1.261	0.103 ± 1.45	0.0003
TSH (mU/L)*	2.44 ± 9.28	1.28 ± 4.21	0.0088
FT4 (ng/dL)	4.97 ± 5.77	4.27 ± 5.56	0.82
Calcium (mg/dL)	9.5 ± 0.66	9.51 ± 0.63	0.58
Phosphorus (mg/dL)	3.70 ± 0.77	3.61 ± 0.71	0.083
Parathormone (ng/dL)	52.4 ± 38.2	48.2 ± 30.3	0.45
25(OH)D (ng/dL)	21.9 ± 11.7	22.9 ± 11.9	0.40
Osteocalcin (ng/mL)	5.91 ± 3.96	5.57 ± 3.53	0.0001
C-telopeptide (ng/mL)	0.47 ± 0.36	0.37 ± 0.22	0.15

* The mean TSH levels during the preceding year.

According to TSH suppression, patients in the severely, moderately, and mildly suppressed and non-suppressed groups experienced at least one VF at rates of 44% (*n* = 8), 15.8% (*n* = 49), 15.6% (*n* = 43), and 25.8% (*n* = 99), respectively. The fracture rate in the severe suppression group was significantly greater than in the moderately and mildly suppressed and non-suppressed groups (*P* = 0.013).

Five patients (27.7%) in the severe suppression group, 29 (9.35%) in the moderate suppression group, 29 (10.5%) in the mild suppression group, and 68 (16.2%) in the non-suppressed group all experienced grade 2 or 3 VFs.

There was no statistically significant difference in the prevalence of VFs between patients who received radioactive iodine therapy and those who did not (79.9 vs 79.8%, *P* = 1.000). Comparison of age, sex, bone densitometry, and VF characteristics between patients who received RAI therapy and those who did not is given in Supplement 3.

In patients with VFs, the mean total number of fractures was 4.45 ± 1.94 (median: 4, range: 1–9). The distribution by severity showed a predominance of mild fractures, with a mean of 3.21 ± 1.32 grade 1 fractures (median: 3, range: 1–7). Moderate fractures (grade 2) were less frequent, with a mean of 1.87 ± 1.21 (median: 2, range: 1–5), while severe fractures (grade 3–4) were relatively uncommon, with a mean of 1.42 ± 0.68 (median: 1, range: 1–3). Regionally, fractures were predominantly located in the thoracic spine, with a mean of 4.12 ± 1.65 fractures (median: 4, range: 1–9), whereas lumbar fractures were less frequent, with a mean of 1.36 ± 0.92 (median: 1, range: 1–4). These findings indicate that VF burden in this cohort is largely driven by multiple, predominantly mild fractures, with a clear predilection for the thoracic region. Detailed data are given in Supplement 4.

Among DTC patients with and without vertebral X-ray, age (51.44 ± 11.81 vs 51.50 ± 11.25 years), levothyroxine dose (1.63 ± 0.41 vs 1.64 ± 0.38 μg/kg/day), BMI (29.7 vs 30.4 kg/m^2^), TSH levels (*P* = 0.447), and disease duration (5.37 ± 4.24 vs 5.05 ± 4.64 years, *P* = 0.06) were comparable between the two groups.

### Assessment of patients according to TSH levels

Table 4 presents the clinical and laboratory characteristics of patients according to TSH suppression levels. Thirty-three percent of patients had TSH levels below 0.1 mIU/L. The duration of the illness varied between groups. The duration of the disease was longer in groups with mild suppression (*P* = 0.006, *P* < 0.001) than in groups with moderate or severe suppression (*P* < 0.001).

**Table 4:** DTC patients’ clinical and laboratory characteristics according to TSH suppression.

	TSH < 0.01	TSH 0.01–0.1	TSH 0.1–0.5	TSH > 0.5	*P*
	(*n* = 18)	(*n* = 310)	(*n* = 275)	(*n* = 387)	
Age (years)	48.5 ± 12	50.3 ± 10.7	51.8 ± 11.8	52.1 ± 11.86	0.11
Duration of disease years)	3.61 ± 3.95	4.16 ± 3.65	5.58 ± 4.22	5.84 ± 5.02	<0.001
BMI (kg/m^2^)	29.4 ± 3.88	29.5 ± 5.31	31.1 ± 5.87	30.9 ± 5.85	0.014
SBP (mmHg)	124.5 ± 15.9	124 ± 19.06	123 ± 18.1	128.3 ± 19.4	0.018
DBP (mm/Hg)	79.8 ± 9.12	77.6 ± 12.1	75.2 ± 11.1	79.4 ± 12	0.003
Pulse (per minute)	81.6 ± 4.94	80.4 ± 9.67	79.4 ± 10.5	78.9 ± 10.4	0.14
LT4 dose μg/kg	1.88 ± 0.81	1.82 ± 0.48	1.65 ± 0.43	1.60 ± 0.45	<0.001
LT4 daily dose (μg/day)	134 ± 56.9	138 ± 36.8	130.1 ± 34.8	125.9 ± 38	<0.001
Femur neck BMD (g/cm^2^)	0.880 ± 0.14	0.940 ± 0.16	0.951 ± 0.18	0.929 ± 0.205	0.61
Femur neck T score	−0.81 ± 1.03	−0.307 ± 1.21	−0.16 ± 1.35	−0.39 ± 1.2	0.21
Femur neck z score	−0.13 ± 0.75	0.289 ± 1.09	0.44 ± 1.21	0.22 ± 1.15	0.16
L1–L4 BMD (g/cm^2^)	1.080 ± 0.14	1.075 ± 0.21	1.080 ± 0.206	1.07 ± 0.19	0.89
L1–L4 T score	−0.62 ± 1.08	−0.43 ± 1.58	−0.44 ± 1.51	−0.44 ± 1.78	0.93
L1–L4 Z score	−0.22 ± 1.1	−0.035 ± 1.5	0.03 ± 1.40	−0.0003 ± 1.4	0.83
Fracture rate *n* (%)	8 (44%)	49 (15.8%)	43 (15.6%)	99 (25.8%)	0.013
TSH (mU/L)	0.005 ± 0.002	0.035 ± 0.025	0.24 ± 0.109	4.78 ± 12.3	<0.001
FT4 (ng/dL)	10.1 ± 8.15	3.18 ± 4.86	3.18 ± 4.78	3.45 ± 4.72	<0.001
Calcium (mg/dL)	9.42 ± 0.65	9.44 ± 0.58	9.49 ± 0.58	9.49 ± 0.614	0.48
Phosphorus (mg/dL)	4.16 ± 0.66	3.53 ± 0.72	3.58 ± 0.72	±0.71	0.002
Parathormone (ng/dL)	39.2 ± 17.5	52.4 ± 34.5	52.9 ± 38.7	51.7 ± 30.4	0.42
25(OH)D (ng/dL)	23.7 ± 11.2	22.07 ± 14.2	22.6 ± 13.6	22.6 ± 11.3	0.34
Osteocalcin (ng/mL)	6.56 ± 4.51	5.77 ± 4.61	4.49 ± 3.28	4.94 ± 4.58	0.064
C-telopeptide (ng/mL)	0.42 ± 0.31	0.43 ± 0.35	0.31 ± 0.12	0.305 ± 0.28	0.0064

Data were presented as mean standard deviation.

The moderate suppression group’s BMI was lower than that of the non-suppressed group (*P* = 0.0056) and mild suppression group (*P* = 0.0061). Daily doses of levothyroxine varied by group (*P* < 0.001). The moderately suppressed group received a greater daily dose of levothyroxine than the non-suppressed group (*P* < 0.001). Based on suppression levels, the patient’s femur neck BMD and L1–L4 BMD levels were comparable among groups.

### Assessment of patients according to the menopausal state

Five hundred and five women were categorized as postmenopausal. Table 5 shows the laboratory analysis of participants grouped by menopause status. Premenopausal and postmenopausal patients had similar TSH levels. L1–L4 and femur neck BMD measurements in the postmenopausal group were lower than those in the premenopausal group (*P* < 0.001). Premenopausal women and men had lower calcium levels, which are essential for bone health, and 25(OH)D levels, which indicate vitamin D status, than postmenopausal women and men (*P* < 0.001).

**Table 5:** Clinical and laboratory characteristics according to sex and menopausal status.

	Premenopausal women (*n* = 323)	Postmenopausal women (*n* = 505)	Men (*n* = 162)	*P*
Age (years)	40.6 ± 7.28	57.7 ± 7.8	53.3 ± 12.5	<0.001
Duration of disease (years)	4.29 ± 3.16	5.9 ± 4.96	4.85 ± 4.64	<0.001
BMI (kg/m^2^)	29.3 ± 6.24	31.7 ± 5.47	28.9 ± 4.22	<0.001
SBP (mmHg)	117.9 ± 14.9	129.2 ± 19.7	129.2 ± 19.2	<0.001
DBP (mm/Hg)	74.7 ± 10.7	79.01 ± 11.9	79.4 ± 12.6	<0.001
Pulse (per minute)	79.6 ± 9.12	79.01 ± 10.2	82 ± 12.3	0.13
LT4 daily dosage (μg/day)	135.4 ± 38.1	121.3 ± 32.1	152.2 ± 41.3	<0.001
Femur neck BMD (g/cm^2^)	0.980 ± 0.208	0.880 ± 0.15	1.000 ± 0.18	<0.001
Femur neck T score	0.11 ± 1.15	−0.61 ± 1.2	−0.23 ± 1.32	<0.001
Femur neck z score	0.33 ± 1.09	0.24 ± 1.11	0.38 ± 1.35	0.08
L1–L4 BMD (g/cm^2^)	1.140 ± 0.198	1.012 ± 0.18	1.130 ± 0.22	<0.001
L1–L4 T score	0.204 ± 1.69	−0.98 ± 1.36	−0.07 ± 1.73	<0.001
L1–L4 Z score	0.11 ± 1.31	−0.14 ± 1.38	0.18 ± 1.75	0.004
Mean TSH within one year (mU/L)	2.45 ± 10.1	1.7 ± 7.31	1.73 ± 4.43	0.84
FT4 (ng/dL)	3.18 ± 4.69	3.55 ± 5.07	3.3 ± 4.87	0.14
Calcium (mg/dL)	9.26 ± 0.63	9.57 ± 0.55	9.6 ± 0.52	<0.001
Phosphorus (mg/dL)	3.56 ± 0.69	3.709 ± 0.73	3.309 ± 0.65	<0.001
Parathormone (ng/dL)	49.6 ± 33	53.2 ± 33.7	53.1 ± 36.8	0.09
25(OH)D (ng/dL)	20.3 ± 13.02	23.07 ± 13	24.8 ± 11.7	<0.001
Osteocalcin (ng/mL)	4.63 ± 4.7	5.51 ± 4.17	4.85 ± 3.59	0.006
C-telopeptide (ng/mL)	0.33 ± 0.205	0.44 ± 0.31	2.59 ± 12.6	0.05

The BMI of the postmenopausal group exhibited a statistically significant increase compared with both the premenopausal women and males (*P* < 0.001). The systolic blood pressure (SBP) (*P* < 0.001) and diastolic blood pressure (DBP) (*P* < 0.001) of the premenopausal group exhibited a statistically significant decrease when compared with both postmenopausal women and males.

No significant association was seen between TSH levels and femur neck and L1–L4 BMD levels in premenopausal (*P* = 0.53, *P* = 0.38) and postmenopausal women (*P* = 0.6, *P* = 0.7) and men (*P* = 0.59, *P* = 0.5). There was no statistically significant link observed between the length of the disease and the levels of BMD in the femur neck and L1–L4 vertebrae (*P* = 0.11, *P* = 0.65) in all groups.

### Analysis of osteoporotic and fracture status variables

In univariate analysis, age, sex, disease duration, mean daily LT4 dose/kg, femur neck BMD, L1–L4 BMD, C-telopeptide, and osteocalcin were significantly associated with osteoporotic status. In multivariate logistic regression analysis, age, femur neck BMD, L1–L4 BMD, and osteocalcin remained independent predictors of osteoporosis. Increasing age and osteocalcin levels were associated with a higher likelihood of osteoporosis, whereas higher femoral neck and L1–L4 BMD values were associated with a lower risk. The overall model was statistically significant (*P* < 0.001, pseudo-*R*^2^: 0.427).

The factors affecting the fracture status were age, sex, duration of disease, mean daily levothyroxine dose/kg, femur neck BMD, L1–L4 BMD, and FT4 levels in univariate analysis. In multivariate logistic regression analysis, disease duration, FT4 levels, and femur neck BMD remained independent predictors of fracture status. Longer disease duration and higher femoral neck BMD were associated with a decreased risk of fractures. The overall model was statistically significant, although the explanatory power was modest (*P* < 0.001, pseudo-*R*^2^: 0.028). The multivariate analysis results are shown in Table 6.

**Table 6:** Multivariate analysis of osteoporotic and fracture status.

Dependent variable		Odds ratio	Standard error	%95 CI	*P*	Pseudo-*R*^2^
Fracture status	Disease duration	1.047	0.0222	1.0046–1.092	0.03	0.028
	FT4	1.051	0.0188	1.014–1.088	0.005	
	Femur neck BMD	0.237	0.156	0.0652–0.866	0.029	
Osteoporotic status	Age	1.0046	0.321	0.374–1.634	0.002	0.427
	Femur neck BMD	−8.472	1.016	−10.553–6.392	<0.001	
	L1–L4 BMD	−9.744	0.979	−11.665–7.824	<0.001	
	Osteocalcin	0.549	0.0276	0.000669–0.109	0.047	

## Discussion

In the present study, we reported 42.0% of the thyroid cancer patients under LT4 treatment exhibited VFs and 27.5% of them had moderate/severe VFs. In multivariate analysis, disease duration, FT4 level, and femur neck BMD emerged as independent determinants of fracture status. Prolonged disease duration and elevated FT4 levels were associated with a higher probability of fractures, suggesting a potential cumulative and metabolic effect on bone fragility. In contrast, higher femoral neck BMD was protective against fractures. Despite the statistical significance of the model (*P* < 0.001), the relatively low pseudo-*R*^2^ indicates that additional factors not included in the model may contribute to fracture risk. TSH levels were not associated with VF. In addition, there was no significant relationship between TSH levels and BMD values.

Although the mean TSH level over the past year was higher in individuals with fractures, we observed that fractures were more frequent in the group with markedly suppressed TSH levels when patients were categorized according to TSH ranges. This seemingly paradoxical finding may reflect heterogeneity within the study population and suggests that average TSH levels alone may not adequately capture fracture risk. It is possible that periods of suppressed TSH, even if not reflected in the annual mean, contribute more significantly to bone fragility. Keeping patients inappropriately in severe suppression degree affects bone negatively. Alternatively, confounding factors or differences in patient characteristics between groups may have influenced these results.

The VF rates we observed are in accordance with earlier European research that found that patients with DTC had greater VF rates than the general population.

Mazziotti *et al.* reported a 28.5% prevalence of radiological VFs, with a fracture prevalence of 44.6% in the TSH < 0.5 mIU/L subgroup among 179 women undergoing TSH suppressive therapy for thyroid carcinoma (13). A small study from Japan revealed that the prevalence of VFs was significantly higher in the DTC group (44.1%) than in the control group (16.3%) (14).

Contrary studies have reported no increased risk of VFs under long-term LT4 suppression in patients with DTC, both among men and women (11, 12).

We observed osteoporosis in 8.2% and osteopenia in 36.6% of the study group, which is compatible with the literature. In a study that enrolled patients with DTC, osteoporosis was observed in 7.3% of patients, which was more frequent according to the control group (5.3%) (4).

The impact of TSH suppression treatment on BMD in patients with DTC remains unclear, with conflicting results reported in the literature. Most cross-sectional studies in the literature report no significant change in BMD levels in premenopausal women compared with the control group (11, 17, 18, 19, 20, 21, 22, 23). However, two meta-analyses indicate detrimental effects on BMD levels in postmenopausal women and men (8, 9).

A large-scale longitudinal investigation found that women over 50 years old undergoing TSH suppression treatment had lower BMD than women with thyroid cancer who had normal TSH levels (24). Cross-sectional studies have also produced contradictory results (4, 6, 25, 26, 27, 28). A study observed no difference in BMD levels between treatment and control groups after 7.3 years of TSH suppression treatment in DTC patients (25).

Chinese women demonstrated a decline in all-site BMD levels after 12.2 years of TSH suppression therapy (26). In our study, we found that disease duration and age are independent risk factors for lower BMD and VFs.

In two large-scale case–control studies from a population database, increased osteoporosis incidence was found in the thyroid cancer population, but fracture incidence was not higher in the patient population compared with the control group, indicating that TSH suppression has a negative effect on bone (7, 10).

The current study possesses significant strengths, having been conducted with a large cohort of DTC patients in a multicenter setting, simultaneously evaluating vertebral X-rays and BMD.

This study has several limitations. VFs are assessed using X-ray imaging. Vertebral X-ray data were accessible for 48% of the research participants. The assessment of VFs using DXA is preferred for evaluating such fractures; however, it was unavailable in Turkey. In addition, the inability to evaluate trabecular bone score constituted one of the limitations of this study. Due to the limited availability of the required software across clinical centers in Turkey, bone quality could not be assessed. Data on potential confounders, such as vitamin D and calcium supplementation, smoking status, alcohol consumption, prior history of osteoporosis, and family history, were not available.

The patients in this study were managed according to ATA 2015 guidelines, and the updated ATA 2025 guidelines do not specify a TSH suppression target based on risk category; it is individualized and re-evaluated over time based on response and balance of benefits and risks. The findings of the present study can be interpreted with the results of studies conducted up to 2025.

The study design was cross-sectional, and there were no age- and sex-matched healthy control groups, but the TSH mean level was calculated for each patient with current, 6-month-ago, and 1-year-ago TSH levels. The exact number of VFs in subjects not receiving LT4 suppression is unknown. A retrospective study indicated a 20.8% VF rate in osteoporotic Turkish women (27), while a cross-sectional study found a 12.1% prevalence of vertebral compression fractures in the Turkish women population (28).

This study indicates that the prevalence of VFs in patients with DTC undergoing levothyroxine suppression is higher than that reported in postmenopausal osteoporotic groups in the literature.

In conclusion, the findings of this study suggest that DTC patients undergoing TSH suppression have a significant risk of VFs. The incidence of VFs correlates with disease duration, FT4, and femur neck BMD in the studied cohort. The incidence of VF was elevated in the group with severe TSH suppression, but mean TSH levels were not found as a contributing factor in multivariate analysis. Periods of suppressed TSH levels or other confounding factors may have influenced these results, such as variations in patient adherence to medication, differences in thyroid hormone replacement therapy, or the presence of other underlying health conditions that could affect TSH levels.

Incorporating VF assessment into the routine care of DTC survivors may help ensure long-term skeletal safety.

## Supplementary materials









## Declaration of interest

The authors declare that there is no conflict of interest that could be perceived as prejudicing the impartiality of the work reported.

## Funding

No grants or fellowships supported the writing of the paper.

## Author contribution statement

DGY planned this multicentric study and reviewed the manuscript. OB and DGY evaluated the vertebral X-rays and BMD. Data collection was done by the remaining contributors. CDY and DGY wrote the manuscript and prepared the database. CI made the analysis of data.

## Data availability

All datasets generated during and/or analyzed during the current study are not publicly available but are available from the corresponding author on reasonable request.

## Patient consent

Consent was obtained from each patient or subject after full explanation of the purpose and nature of all procedures used.

## Artificial intelligence

Artificial intelligence was not used in this article.

## Ethical approval

This study was approved by Marmara University Local Ethics Committee with protocol number 09.2017.359.
