# Supplementary material for: Vertebral fracture risk in patients with differentiated thyroid cancer receiving TSH-suppressive therapy
Source: Endocr Connect. 2026 Jul 17;15(7):e260103. doi: 10.1530/EC-26-0103 (PMC13386147; doi:10.1530/EC-26-0103)
Supplement: Supplementary file 1 [file EC-26-0103_supplement_1.pdf]

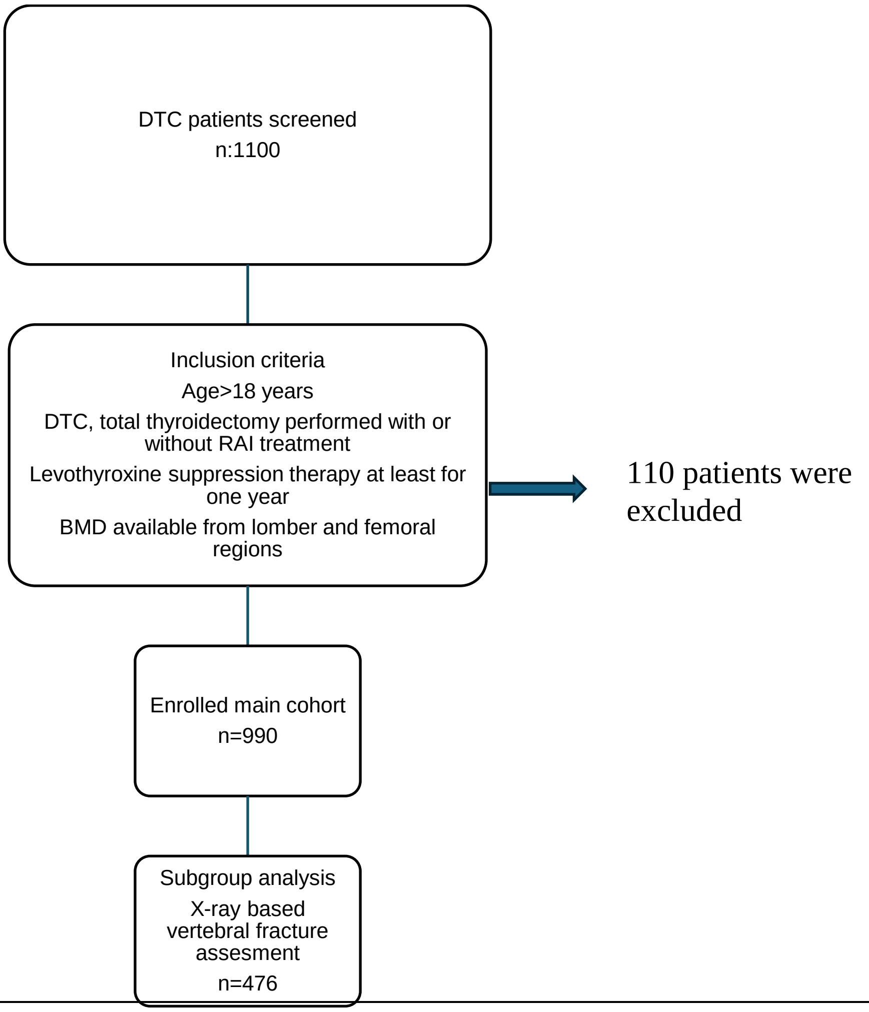

Figure 1Patient selection diagram (supplement)

DTC: differentiated thyroid carcinoma, BMD: bone mineral density
