# Supplementary material for: Vertebral fracture risk in patients with differentiated thyroid cancer receiving TSH-suppressive therapy
Source: Endocr Connect. 2026 Jul 17;15(7):e260103. doi: 10.1530/EC-26-0103 (PMC13386147; doi:10.1530/EC-26-0103)
Supplement: Supplementary file 2 [file EC-26-0103_supplement_2.pdf]

Supplement 2 : . Distribution of thyroid cancer and RAI characteristics by sex

Characteristic	Total (n=990)	Female (n=829)	Male (n=161)	p
**Thyroid cancer type**				
– Papillary	968 (97.7%)	813(98.06%)	155 (96.2%)	0.062
– Follicular	20 (2.02%	14 (1.76%)	6 (3.72%)	
– Other	2 (0.2%)	2 (0.25%)	0 (0%)	
**Thyroid cancer subtype**				
– Classic papillary	492 (49.6%)	427 (51.5%)	65 (40.3%)	0.959
– Follicular variant	390 (39.3%)	318 (38.3%)	72 (44.7%)	
– Tall cell / oncocytic / other	108 (10.9%)	84 (10.1%)	24 (14.9%)	
**Lymph node metastasis**				
– None	809 (81.7%)	683 (82.4%)	126 (78.3%)	**0.024**
**Tumor stage**				
– Stage I	939 (94.8%)	794 (95.8%)	145 (90.1%)	**0.003**
– Stage II	29 (2.9%)	19 (2.3%)	10 (6.2%)	
– Stage III	14 (1.4%)	12 (1.4%)	2 (1.2%)	
– Stage IV	8 (0.8%)	4 (0.5%)	4 (2.5%)	
Patients receiving RAI therapy, n (%)	593 (59.9%)	475 (57.3%)	118 (73.3%)	**<0.001**

Data are presented as n (%) for categorical variables p values: Chi-square test for categorical variables
