# Supplementary material for: Vertebral fracture risk in patients with differentiated thyroid cancer receiving TSH-suppressive therapy
Source: Endocr Connect. 2026 Jul 17;15(7):e260103. doi: 10.1530/EC-26-0103 (PMC13386147; doi:10.1530/EC-26-0103)
Supplement: Supplementary file 3 [file EC-26-0103_supplement_3.pdf]

Supplement 3: Comparison of age, sex, bone densitometry and vertebral fracture characteristics between patients who received RAI therapy and those who did not

Characteristic	RAI (n=593)	No RAI (n=383)	p
Age (years), median (IQR)	51.0 (42.2–60.0)	52.0 (45.0–59.0)	0.764
**Sex**			
– Female	475 (80.1%)	342 (89.3%)	**<0.001**
– Male	118 (19.9%)	41 (10.7%)	
**Bone mineral density assessment**			
– Normal	333 (56.2%)	204 (53.3%)	0.653
– Osteopenia	211 (35.6%)	147 (38.4%)	
– Osteoporosis	49 (8.3%)	32 (8.4%)	
Femoral neck BMD (g/cm²), median (IQR)	0.929 (0.837–1.029)	0.920 (0.817–1.035)	0.419
Femoral neck T-score, median (IQR)	-0.50 (-1.10–0.40)	-0.40 (-1.20–0.60)	0.751
Femoral neck Z-score, median (IQR)	0.20 (-0.50–0.95)	0.20 (-0.40–1.00)	0.982
L1–4 BMD (g/cm²), median (IQR)	1.074 (0.948–1.203)	1.055 (0.937–1.195)	0.433
L1–4 T-score, median (IQR)	-0.50 (-1.50–0.50)	-0.70 (-1.60–0.40)	0.306
L1–4 Z-score, median (IQR)	-0.20 (-1.00–0.80)	-0.20 (-0.90–0.70)	0.873
**Vertebral fracture status**			
– Present	227 (38.3%)	146 (38.1%)	0.998
– Absent	57 (9.6%)	37 (9.7%)	
– Unknown	308 (51.9%)	200 (52.2%)	
**Among patients with vertebral fracture (RAI n=227; No RAI n=146)**			
– Total fracture count, median (IQR)	4.00 (3.00–6.00)	4.00 (3.00–6.00)	0.367
– Grade 1 fractures present	217 (95.6%)	136 (93.2%)	
– Grade ≥2 fractures present	127 (55.9%)	70 (47.9%)	
– Lumbar fractures present	70 (30.8%)	45 (30.8%)	
– Thoracic fractures present	216 (95.2%)	143 (97.9%)	

Data are presented as n (%) for categorical variables and median (IQR: interquartile range) for continuous variables.

p values: Chi-square (χ^2) test for categorical variables; Mann-Whitney U test (MWU) for continuous variables.

BMD: Bone mineral density. RAI: Radioactive iodine therapy. Vertebral fracture subgroup analyses are restricted to patients with confirmed vertebral fractures (RAI n=227; No RAI n=146). Grade ≥2 fractures include moderate and severe morphometric fractures. Missing data were excluded from each analysis.
