# Supplementary material for: Vertebral fracture risk in patients with differentiated thyroid cancer receiving TSH-suppressive therapy
Source: Endocr Connect. 2026 Jul 17;15(7):e260103. doi: 10.1530/EC-26-0103 (PMC13386147; doi:10.1530/EC-26-0103)
Supplement: Supplementary file 4 [file EC-26-0103_supplement_4.pdf]

**Supplement 4: Distribution of Vertebral Fracture Counts by Grade and Anatomical Region**

| Variable | Value (Mean ± SD; Median [Min–Max]) |
|---|---|
| **Total number of fractures** | 4.45 ± 1.94<br>4 (1–9) |
| **Grade 1 fractures** | 3.21 ± 1.32<br>3 (1–7) |
| **Grade 2 fractures** | 1.87 ± 1.21<br>2 (1–5) |
| **Grade 3–4 fractures** | 1.42 ± 0.68<br>1 (1–3) |
| **Lumbar fractures** | 1.36 ± 0.92<br>1 (1–4) |
| **Thoracic fractures** | 4.12 ± 1.65<br>4 (1–9) |
